# The role of surgery and radiation in advanced gastric cancer: A population-based study of Surveillance, Epidemiology, and End Results database

**DOI:** 10.1371/journal.pone.0213596

**Published:** 2019-03-12

**Authors:** Shuang Ye, Lu Wang, Zhigang Zuo, Yanping Bei, Kaitai Liu

**Affiliations:** 1 Department of Radiation Oncology, Lihuili Hospital, Ningbo Medical Center, Ningbo, Zhejiang, China; 2 Department of Radiation Oncology, Shanghai Tenth People’s Hospital, Tongji University School of Medicine, Shanghai, China; 3 Department of Radiation Oncology, Shiyan People’s Hospital, Hubei University of Medicine, Shiyan, Hubei, China; VA Boston Healthcare System, Harvard Medical School (Brigham and Women's Hospital), UNITED STATES

## Abstract

**Background:**

Chemotherapy is the standard approach for advanced gastric cancer, while the role of local therapy such as surgery and radiation for this population remains controversial. Our purpose is to evaluate the effect of local therapies on cancer specific survival (CSS) for advanced gastric cancer patients.

**Methods:**

Four subgroups of patients in different treatment strategies: surgery, radiation (RT), surgery and radiation (Surgery+RT), no surgery/no radiation (No Surgery/No RT) were identified from the Surveillance, Epidemiology, and End Results (SEER)-registered database. The risk factors and the survival outcomes were analyzed by multivariable Cox regression models and Kaplan-Meier methods.

**Results:**

A total of 10,354 patients were eligible with 6658 males and 3696 females. The 5-year CSS in the four subgroups of “Surgery”, “RT”, “Surgery+RT” and “No Surgery/No RT” were respectively 8.9%. 5.7%, 19.8% and 3.2%, which were significantly different in multivariate Cox regression (P<0.001) and univariate log-rank test (P<0.001). Advanced stage categories were defined as stage I, II and III of T/N category according to different initial T and N status following American Joint Committee on Cancer (AJCC) staging principle. Further analysis showed that patients in the group of “Surgery+RT” have significant benefits of survival specifically on stage II and III of T/N category. “Surgery+RT” group and “Surgery” group patients have similar survival time in stage I of T/N category. Moreover, we also found CSS benefits from the administration of “Surgery+RT” in the patients aged both ≥75 and <75 years. Remarkably, patients in “Surgery” group have no different survival time with “RT” group in age category of 75 years and older.

**Conclusions:**

Local therapies, including surgery, radiation, and combination of both might associate to improve survival in advanced gastric cancer patients, but confounding due to disease extent and physical status cannot be excluded.

## Introduction

In the past few decades, there has been a great decrease on the gastric cancer incidence all over the world [1]. Nevertheless, the problem of poor prognosis still exists in gastric cancers which is the 3rd cause to cancerous deaths in the world [2]. The diagnosis of gastric cancer is general in IV stage with metastatic disease, and the prognosis is usually poor [3–4]. Studies showed that with no treatment, the median survival of advanced gastric cancer patients was just three to five months, and which could be extended to approximately ten months with palliative chemotherapy [5–6]. Postoperative concurrent chemoradiation has been used to be the standard treatment for locally advanced gastric cancer patients [7–8]. There are several types of treatment strategies for advanced gastric cancer, such as radiotherapy, chemotherapy and palliative gastrectomy [9]. Chemotherapy can relief symptoms as well as enhance life quality and survival for advanced gastric cancer patients [10–11]. Until now, there were conflicting evidences on effects in patients with advanced gastric cancer. External-beam RT as a single modality shows minimal value to advanced gastric cancer patients and does not improve survival [12]. A population-based Surveillance, Epidemiology, and End Results (SEER) analysis showed that only modest improvements in prognosis for metastatic gastric cancer were observed in patients who underwent and in patients who did not undergo palliative gastrectomy [13]. However, patients with stage IV gastric cancer are a heterogeneous group and the role of local therapies for this population remain controversial.

We conducted this population based respective study to evaluate the effect of the local therapies in four sub groups of advanced gastric cancer patients treated with different treatment strategies, including surgery, radiation (RT), surgery and radiation (Surgery+RT), and no surgery/no radiation (No Surgery/No RT) by using the SEER-registered database.

## Materials and methods

### Patients selection

The SEER database was publicly available for studies of cancer-based epidemiology and survival analysis. Our study collected data from 18 population-based cancer registries which included in National Cancer Institute’s (NCI) SEER program. The SEER data are publicly available for studies of cancer-based epidemiology and survival analysis. Since no personal identifying information was used in the analysis and there was no interaction with human subjects, the Institutional Review Board of the Lihuili Hospital, Ningbo Medical Center has granted an exemption to this study.

We selected the gastric cancer cases (C16.0–16.9) whose diagnosis occurred between 2004 and 2013 provided by SEER database (SEER*Stat 8.3.4) in accordance with the classification of site recode. The reason why we chose cases in this period is that the availability of American Joint Committee on Cancer (AJCC) TNM since 2004, and we excluded patients diagnosed after 2013 to make sure a proper follow-up time. We restaged all selected cases based on the instructions provided in the 7^th^ edition staging manual (2010) of AJCC. Histological types were confined only to signet ring cell carcinoma (ICD-03, 8490/3), mucinous adenocarcinoma (ICD-03, 8480/3, 8481/3) and adenocarcinoma (ICD-03, 8140/3, 8144/3, 8210/3, 8211/3, 8221/3, 8255/3, 8260/3, 8261/3, 8262/3, 8263/3, 8310/3, 8323/3). This study has only included stage “IV” patients (any T and/or any N, distant metastases M1). We defined “Stage of T/N” as various advanced stage category according to different initial T and N status following AJCC staging principle. Stage I of T/N included T1N0-1, T2N0; Stage II of T/N included T1N2-3, T2N1-2, T3N0-1, T4aN0; Stage III of T/N included T2N3, T3N2-3, T4aN1-3, T4bN0-3. Only patients older than 18 were included in the study. Patients with any one of the following criteria were also excluded: not the first tumor, unknown treatment, unknown survival time and TNM stage.

All the data used in our study came from the publicly available SEER database with permission granted to access these research data (SEER*Stat username: liuk).

### Statistical analysis

From the database, we extracted the basic information of all selected patients, including gender, race and age, as well as their disease-related information such as CSS, histological type and pathological grading. CSS was calculated from the date of diagnosis to the date of cancer-specific death. Cases died particularly of gastric cancer were taken as events, and those who died for other reasons were taken as censored observations. The chi-square test was used to compare the clinic pathologic variable among various groups. Kaplan-Meier method was used for the survival analysis [14]. A log-rank test was conducted to evaluate the association between specifically prognostic factors and estimated CSS [15]. Cox regression models were used to perform the multi-variate analysis [16]. All models were adjusted for sex (male, female), age (<75, ≥75), race (white, black, other), treatment pattern (surgery, RT, surgery+RT, no surgery/no RT), stage of T/N (stage I, stage II, stage III), pathological grade (I, II, III, IV) and histological type (adenocarcinoma, signet ring cell, mucinous). The statistical test was two sided and P < 0.05 was considered statistically significant. The statistical analyses were conducted by using PASW Statistics 19 (SPSS Inc., Chicago, USA).

## Results

### Patient characteristics and treatment pattern features

A total of 10,354 patients were eligible and the numbers in four different groups of “Surgery”, “RT”, “Surgery+RT”, and “No Surgery/No RT” were 2708, 1355, 777 and 5514 respectively. The age at diagnosis ranged from 13 to 100 years, with a median age of 66 year. Male patients accounting for 64.3% and female patients leaving the left 35.7%. The major race was white (71.2%). Table 1 has presented the summary of the pathological and demographical characteristics of patients. As shown in S1 Fig, stage IV patients at diagnosis accounted for about 30% of the total gastric cancer population, which remained stable between the year 2004 and 2013. In comparison to the noteworthy decrease on “Surgery+RT” and “Surgery” patterns, the “No Surgery/No RT” pattern impressively increased among advanced gastric cancer patients. Moreover, we observed the pattern of “RT” increased in a certain extent from 2004 to 2013, although it was slightly used in only 10% of advanced gastric cancer patients (S2 Fig).

**Table 1:** Patient characteristics.

	Total	Surgery	RT	Surgery+RT	No surgery/No RT	
Variable	n = 10354	n = 2708(%)	n = 1355(%)	n = 777(%)	n = 5514(%)	P value
Sex						<0.001
Male	6658	1628(60.1)	1016(75.0)	531(68.3)	3483(63.2)	
Female	3696	1080(39.9)	339(25.0)	246(31.7)	2031(36.8)	
Age(year)						<0.001
<75	7466	1858(68.6)	1032(76.2)	670(86.2)	3906(70.8)	
≥75	2888	850(31.4)	323(23.8)	107(13.8)	1608(29.2)	
Race						<0.001
White	7372	1822(67.3)	1057(78.0)	531(68.3)	3962(71.9)	
Black	1460	380(14.0)	148(10.9)	99(12.7)	833(15.1)	
Other	1522	506(18.7)	150(11.1)	147(18.9)	719(13.0)	
Pathological grading						<0.001
Grade I	188	37(1.4)	36(2.7)	13(1.7)	102(1.8)	
Grade II	2013	498(18.4)	343(25.3)	125(16.1)	1047(19.0)	
Grade III	6393	1972(72.8)	719(53.1)	562(72.3)	3140(56.9)	
Grade IV	197	80(3.0)	23(1.7)	31(4.0)	63(1.1)	
Unknown	1563	121(4.5)	234(17.3)	46(5.9)	1162(21.1)	
Stage of T/N^a^						<0.001
Stage I	2834	132(4.9)	434(32.0)	30(3.9)	2238(40.6)	
Stage II	2031	463(17.1)	365(26.9)	117(15.1)	1086(19.7)	
Stage III	5489	2113(78.0)	556(41.0)	630(81.1)	2190(39.7)	
Histological type						<0.001
Adenocarcinoma	7326	1768(65.3)	1107(81.7)	505(65.0)	3946(71.6)	
Signet ring cell	252	96(3.5)	24(1.8)	28(3.6)	104(1.9)	
Mucinous	2776	844(31.2)	224(16.5)	244(31.4)	1464(26.6)	

RT, radiation.

^a^ Stage of T/N: Stage I include T1N0-1, T2N0; Stage II include T1N2-3, T2N1-2, T3N0-1, T4aN0; Stage III include T2N3, T3N2-3, T4aN1-3, T4bN0-3.

### Prognostic impacts of various treatment strategies in patients with advanced gastric cancer

**Fig 1:**
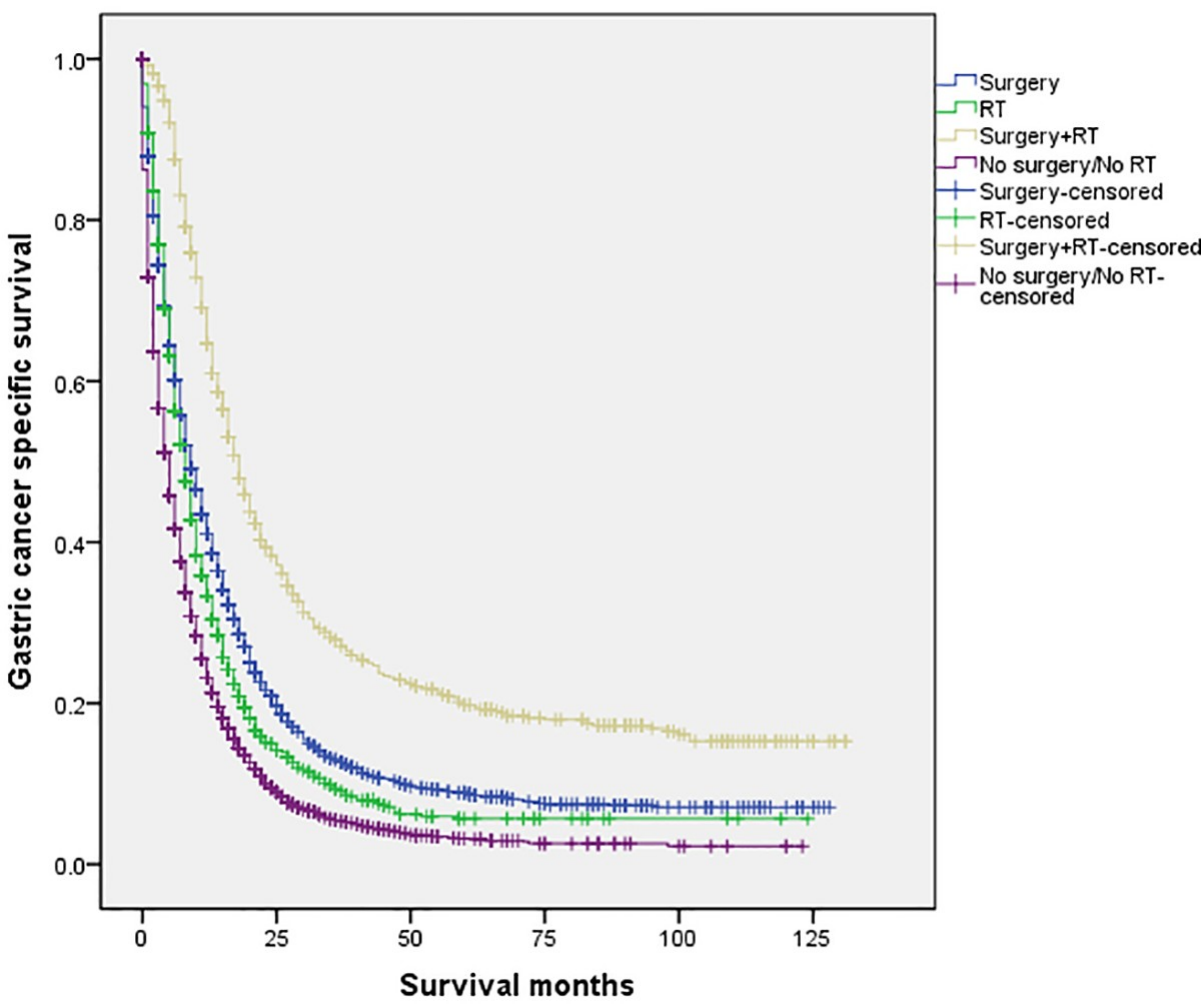
Survival curves in patients with advanced gastric cancer according to four subgroups. RT: radiation.

**Fig 2:**
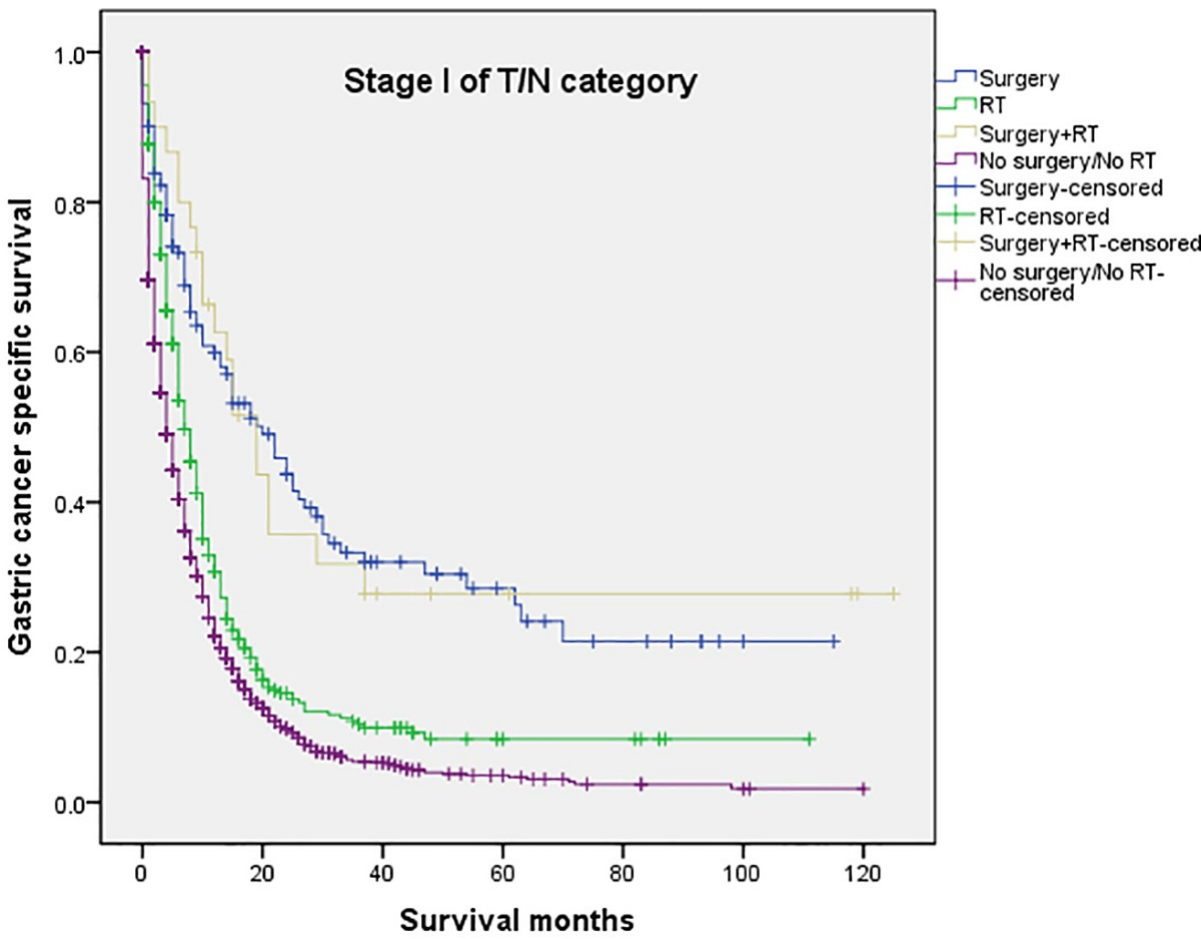
Survival curves in patients with advanced gastric cancer according to four subgroups in stage I of T/N category (P<0.001). RT: radiation.

**Fig 3:**
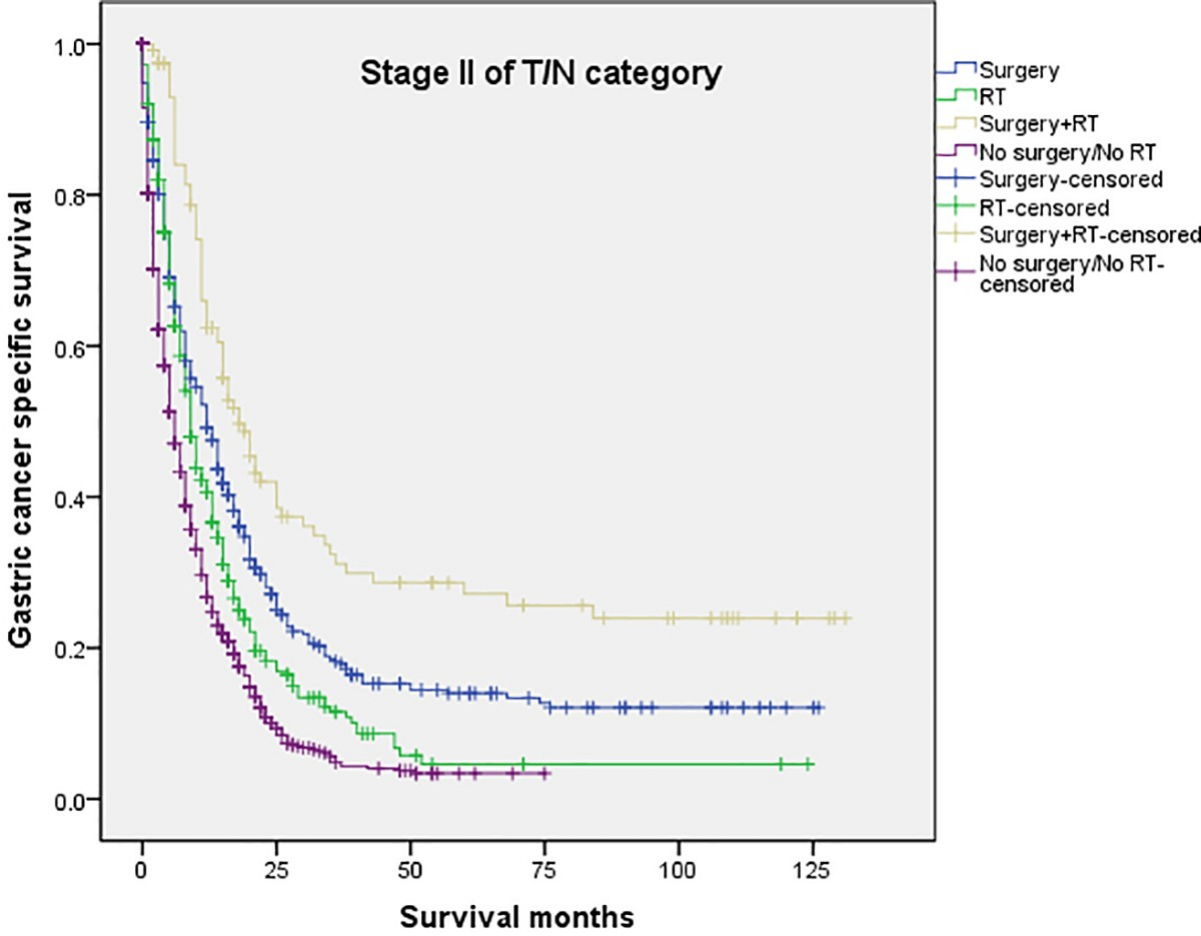
Survival curves in patients with advanced gastric cancer according to four subgroups in stage II of T/N category (P<0.001). RT: radiation.

**Fig 4:**
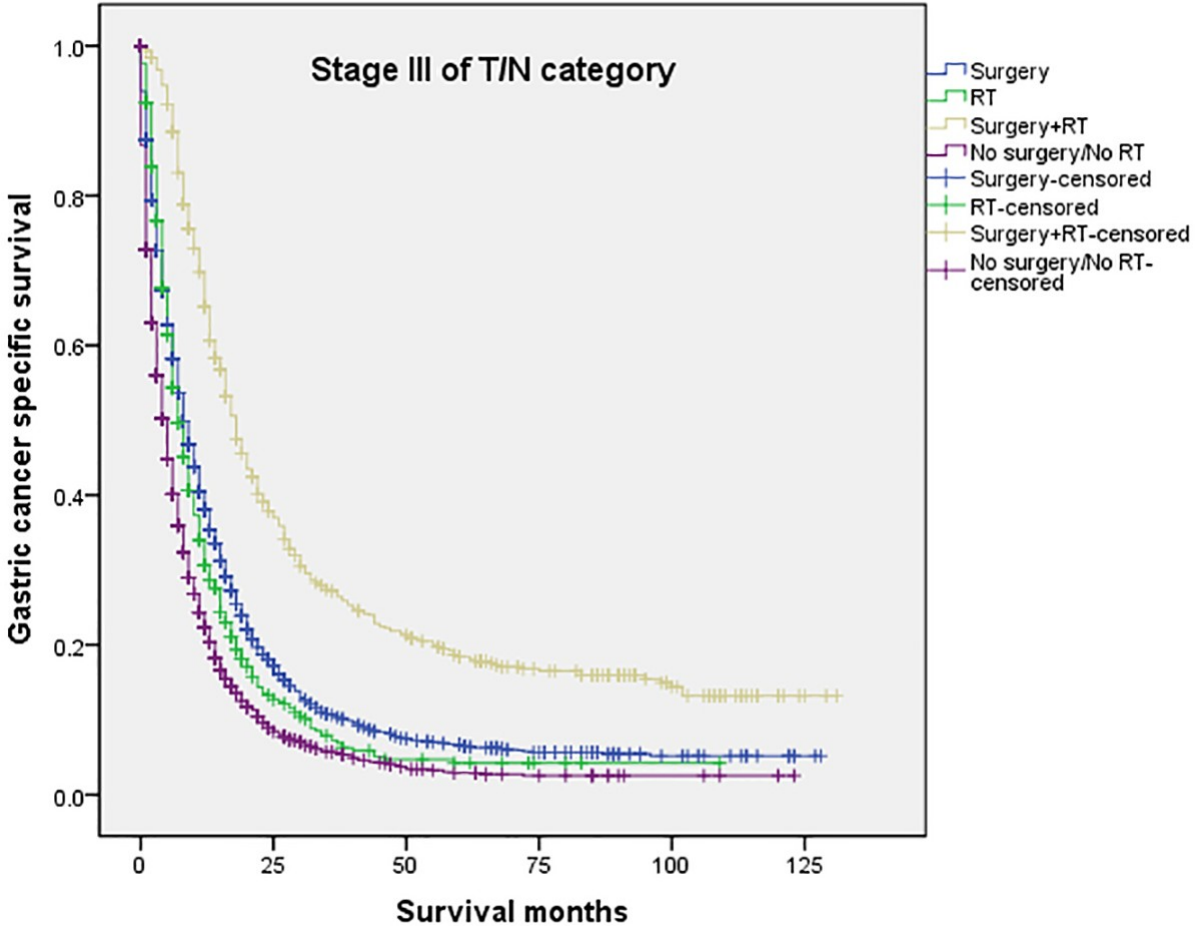
Survival curves in patients with advanced gastric cancer according to four subgroups in stage III of T/N category (P<0.001). RT: radiation.

**Fig 5:**
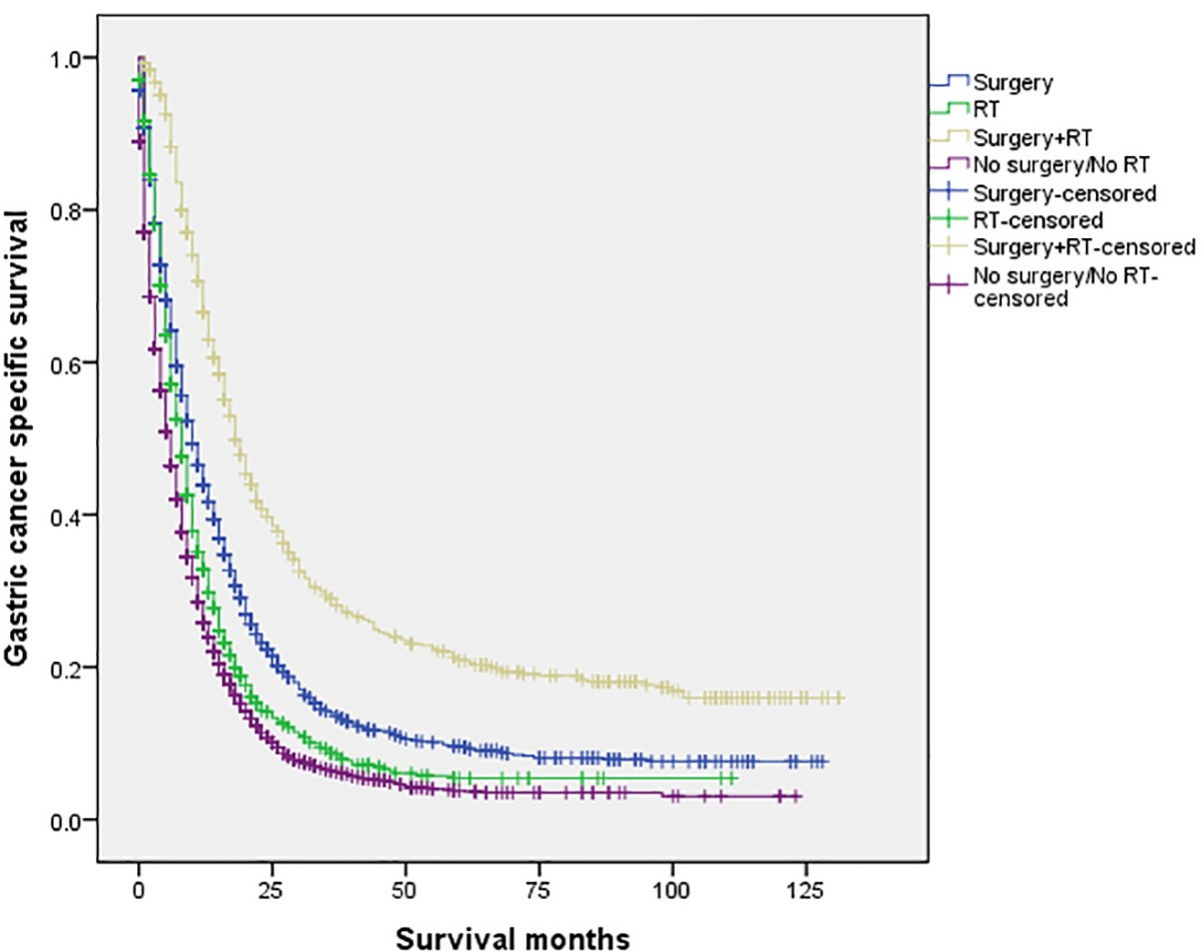
Survival curves in patients with advanced gastric cancer according to four subgroups in age<75 years (P<0.001). RT: radiation.

**Fig 6:**
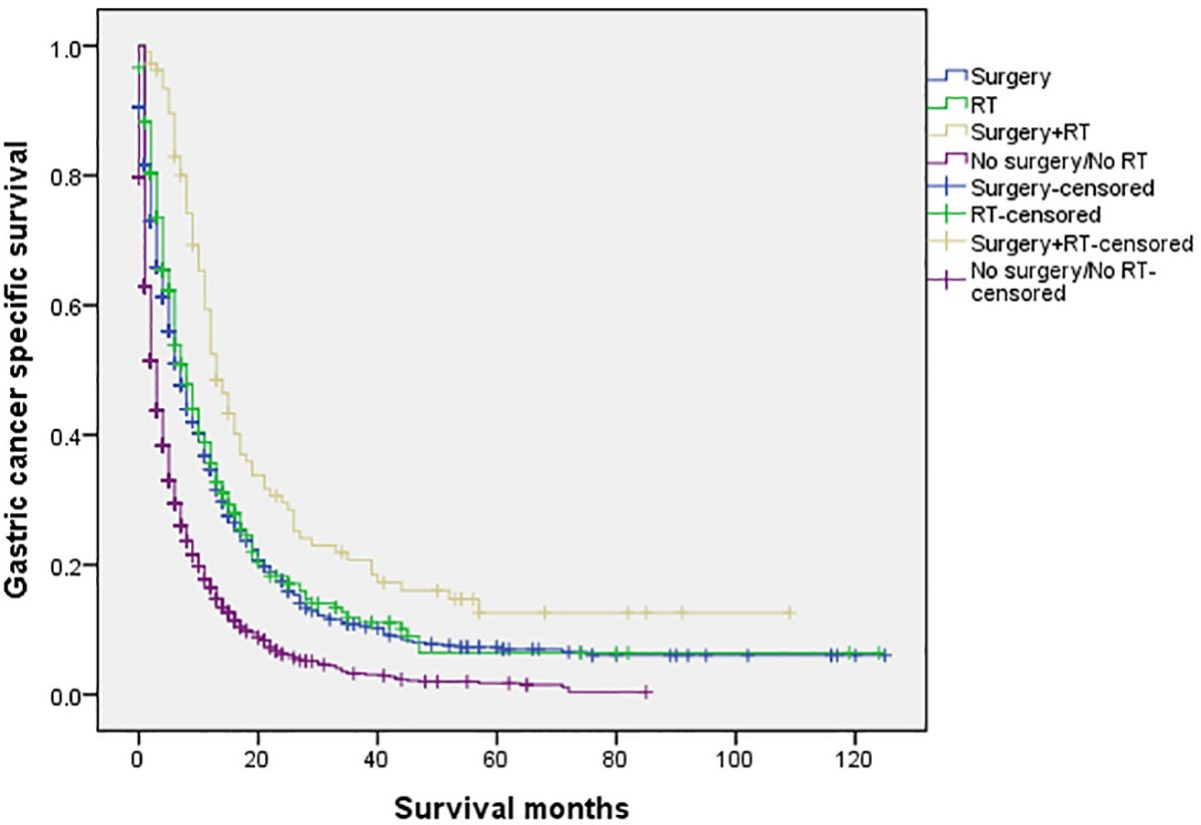
Survival curves in patients with advanced gastric cancer according to four subgroups in age≥75 years (P<0.001). RT: radiation.

The 5-year CSS in the four subgroups of “Surgery”, “RT”, “Surgery+RT” and “No Surgery/No RT” were respectively 8.9%. 5.7%, 19.8% and 3.2%, which were significantly different in univariate log-rank test (P<0.001) (Fig 1). Moreover, the survival analysis was stratified by different stages of T/N category (stage I, II and III, Figs 2–4). It demonstrated that “Surgery+RT” significantly improve cancer specific survival in stage II and III of T/N category (all P<0.001). It was noteworthy that “Surgery+RT” group had no advantage in term of CSS compared with “Surgery” group in stage I of T/N category. Moreover, the survival analysis was also stratified by different age groups (age <75 and ≥75, Figs 5 and 6). It showed that surgery and radiation significantly increased the cancer specific survival for the patients at various age groups (all P < 0.001). Remarkably, patients in “Surgery” group have no different survival time with “RT” group in age category of 75 years and older. Additionally, in the univariate analysis, advanced T/N stages, black and white races, signet ring cancer were identified as the adverse prognostic factors (all P<0.001). Multivariate analyses with cox regression confirmed these factors as independent prognostic factors (Table 2).

**Table 2:** Univariate and multivariate survival analyses of patients with advanced gastric cancer according to various clinicopathological variables.

Variable	n	5-year	Univariate	Multivariate	HR	95% CI
		CSS (%)	P value	P value		
Sex			0.084	0.398		
Male	6658	6.9		0.398	1.020	0.974–1.067
Female	3696	6.1		ref	ref	ref
Age(year)			<0.001	<0.001		
<75	7466	7.4		<0.001	0.731	0.697–0.768
≥75	2888	4.6		ref	ref	ref
Race			<0.001	0.002		
White	7372	6.6		0.100	1.053	0.990–1.120
Black	1460	6.1		0.001	1.148	1.060–1.243
Other	1522	7.4		ref	ref	ref
Pathological grading			<0.001	<0.001		
Grade I	188	7.8		0.051	0.845	0.713–1.001
Grade II	2013	9.6		<0.001	0.824	0.763–0.889
Grade III	6393	6.1		0.722	1.011	0.950–1.076
Grade IV	197	7.8		0.879	1.013	0.859–1.194
Unknown	1563	4.7		ref	ref	ref
Stage of T/N^a^			<0.001	<0.001		
Stage I	2834	8.2		0.105	0.965	0.906–1.009
Stage II	2031	6.6		<0.001	0.834	0.787–0.884
Stage III	5489	6.1		ref	ref	ref
Histological type			<0.001	<0.001		
Adenocarcinoma	7326	7.6		0.816	1.017	0.882–1.172
Signet ring cell	252	4.0		0.107	1.128	0.974–1.305
Mucinous	2776	8.3		ref	ref	ref
Treatment pattern			<0.001	<0.001		
Surgery	2708	8.9		<0.001	0.603	0.570–0.637
RT	1355	5.7		<0.001	0.740	0.692–0.791
Surgery+RT	777	19.8		<0.001	0.366	0.334–0.401
No surgery/No RT	5514	3.2		ref	ref	ref

RT, radiation; CSS, cancer specific survival; HR, hazard ratio; CI, confidence interval; ref, reference.

^a^ Stage of T/N: Stage I include T1N0-1, T2N0; Stage II include T1N2-3, T2N1-2, T3N0-1, T4aN0; Stage III include T2N3, T3N2-3, T4aN1-3, T4bN0-3.

## Discussion

There are high incidents of gastric cancer in lots of nations and regions worldwide. By estimate of the National Cancer Institute’s Surveillance, the number of diagnosed gastric cancer cases in the year of 2016 was about 26,370, and about 10,730 of the patients were estimated to have died of gastric cancer [17]. Historical analysis indicated that there were 34% of the patients whose cancer cells had already spread to other sites when they were first diagnosed with cancer [18], which implies that approximately 30% patients were diagnosed with advanced gastric cancer. Thus, it is important to build standard treatment strategies for them. Similarly, according to our findings, stage IV patients accounted for around 30% of the total number of gastric cancer population, and this ratio remained stable from 2004 to 2013. As we known, because of the heterogeneous extent of primary sites, different degree of disease progression, and patient performance status, it is highly hard to perform prospective randomized controlled trials for patients with advanced gastric cancer. Hence, it is crucial to conduct retrospective analysis for clinical studies on advanced gastric cancer. Our study showed that surgery and radiation might associate with survival improvement in advanced gastric cancer patients according to specifical extent of primary disease and performance status. As far as we know, this study is the biggest population-based retrospective study for the evaluation of the recommended treatment strategies in advanced gastric cancer.

Chemotherapy has been the standard treatment for patients with advanced gastric cancer because of their bad prognosis with incurable complications, like the metastasis in lymph, liver or peritoneum [19–20]. We have reported that appropriate local therapy showed adequate association with the improvement of prognosis in advanced rectal cancer and NSCLC [21–22]. However, the effects of local therapies on advanced gastric cancer patients remain debatable and complex. Hartgrink and colleagues reported that palliative gastrectomy has brought survival improvements to patients aged 70 years and below with a single site of metastasis [19]. Sun and colleagues systematically reviewed studies published previously and represented 3,003 stage IV gastric cancer patients. They found palliative gastrectomy had significant effect on improving the survival time of this group of patients [23]. Another meta-analysis which comprised 19 non-random studies with 2,911 stage IV gastric patients. Results showed that in comparison to those patients who did not receive resection, those who received gastrectomy presented significance improvement in one-year overall survival [24]. In contrast, according to the investigation of REGATTA trials, pre-chemotherapy gastrectomy had little effect on improving the survival time for patients with advanced gastric cancer [25]. Nevertheless, in the subset analysis, distal gastrectomy may have effect on improving the survival time for patients with distal cancers, which remains to be further analyzed. Ito S et al. conducted a retrospective study on the effect of adjuvant surgery after chemotherapy on stage IV gastric cancer patients. They found the rate of three-year OS among the patients who had received adjuvant surgery was 65.6%, and patients who hadn’t was 7.7% (p<0.0001) [26]. Another study also showed that adjuvant surgery had effect on improving the survival for patients with peritoneal washings positive alone [27]. With respect to radiation, there were few studies and inconsistent results. Previous study showed that external-beam RT as a single modality is minimally valuable to advanced gastric cancer patients and does not improve survival [14]. According to the results reported by Strauss and colleagues, adjuvant therapy had significant effect on the improvement of the survival time for the patients at stage III as well as stage IV aged 65 and older [28]. Additionally, they noticed the trend towards the improvement of survival for patients at all ages except for the category of 80 to 85 years. Practically, radiotherapy has another purpose which is to reduce and alleviate the symptoms of disease, like pain, bleeding and stenosis. Study showed that radiotherapy had response rate of 73% patients with advanced gastric cancer, and authors also reported that 30Gy radiation in 10 fractions was an appropriate scheme to treat bleeding symptoms [29].

Our study also showed that the local therapies, including surgery, radiation, and combination of both might have associations with improvement of prognosis for the patients diagnosed with stage IV gastric cancer. Although “Surgery+RT” group seemed to have a better prognosis, it was noteworthy that “Surgery+RT” had no advantage in term of CSS compared with “Surgery” in stage I of T/N category. In our opinion, for advanced gastric cancer patients with early stage of primary mass, the combined therapy may be too invasive due to the ordinary status of performance and poor intrinsic prognostic factors. Furthermore, we found radiation as a local therapy increase survival compared with no radiation/no surgery, patients in surgery group have no different survival with radiation group in age category of 75 years and older, indicating that radiation could be a preferred treatment for older patients who were psychologically or medically unsuitable for surgery. Moreover, we found CSS benefits from the administration of surgery and/or RT at all age categories. Data showed that the average age at diagnosis of gastric cancer was 71–75 years and almost two-thirds of those were above 65 in the USA in 2015 [30–31]. To our minds, local therapies such as surgery and radiation may also be useful for elderly advanced gastric cancer patients. Our results showed that there are rare applications of local therapies, including surgery, radiation and combination of both in advanced gastric cancer in recent ten years which deserves clinical attention.

There are some limitations in our study that deserve mention. First, there are some other relevant information not included in the SEER database, such as the performance status, nutritional status and comorbidities. For example, elderly patients may undergo less aggressive treatments may be due to comorbidities and to poor performance status. Therefore, our results could be confounded due to the non-adjustability of these confounding factors. Secondly, deficiencies of data in radiation technologies and dose, surgery curability and chemotherapy scheme may have resulted in possible significant bias. Third, analysis of a nonrandomized patient population may introduce selection bias. Nonetheless, considering the large population enrolled, this study is still considerably convincing.

## Conclusions

Surgery and radiation might associate with survival improvement in patients with advanced gastric cancer, but confounding due to disease extent and physical status cannot be excluded. However, further prospective studies are required for verification since the study we conducted is a retrospective analysis.

## Supporting information

S1 Fig Trend of the proportion of patients with advanced gastric cancer from 2004 to 2013. (TIF)

S2 Fig Treatment patterns for patients with advanced gastric cancer from 2004 to 2013 according to treatment modality. RT: radiation.
(TIF)

## Abbreviations

CSS: cancer specific survival
SEER: Surveillance, Epidemiology, and End Results
RT: radiation
