# Supplementary figures and images for: The role of surgery and radiation in advanced gastric cancer: A population-based study of Surveillance, Epidemiology, and End Results database
Source: PLoS One. 2019 Mar 12;14(3):e0213596. doi: 10.1371/journal.pone.0213596 (PMC6413929; doi:10.1371/journal.pone.0213596)

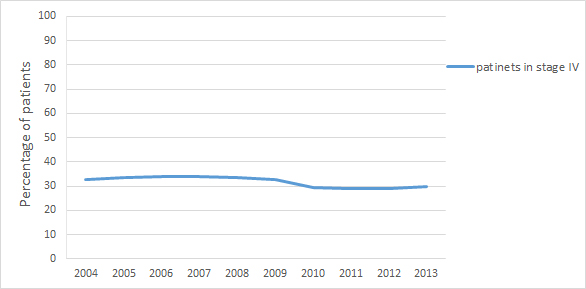

Supplement: S1 Fig — (TIF) [file pone.0213596.s001.tif]

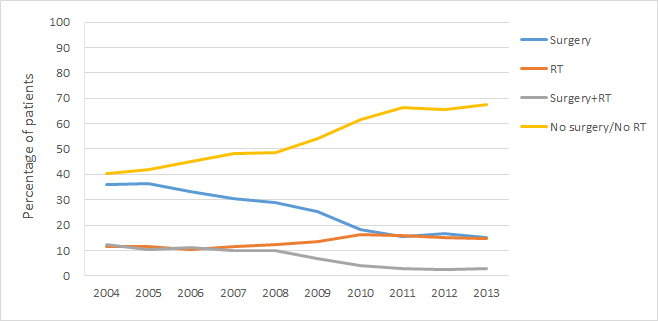

Supplement: S2 Fig — RT: radiation. (TIF) [file pone.0213596.s002.tif]
